# 5-*O*-(*N*-Boc-l-Alanine)-Renieramycin T Induces Cancer Stem Cell Apoptosis via Targeting Akt Signaling

**DOI:** 10.3390/md20040235

**Published:** 2022-03-29

**Authors:** Darinthip Suksamai, Satapat Racha, Nicharat Sriratanasak, Chatchai Chaotham, Kanokpol Aphicho, Aye Chan Khine Lin, Chaisak Chansriniyom, Khanit Suwanborirux, Supakarn Chamni, Pithi Chanvorachote

**Affiliations:** 1 Center of Excellence in Cancer Cell and Molecular Biology, Faculty of Pharmaceutical Sciences, Chulalongkorn University, Bangkok 10330, Thailand; 6272002733@student.chula.ac.th (D.S.); nicharat.s@alumni.chula.ac.th (N.S.); chatchai.c@chula.ac.th (C.C.); 2 Graduate Program of Pharmaceutical Science and Technology, Faculty of Pharmaceutical Science, Chulalongkorn University, Bangkok 10330, Thailand; 6373015133@student.chula.ac.th; 3 Interdisciplinary Program in Pharmacology, Graduate School, Chulalongkorn University, Bangkok 10330, Thailand; 6481007020@student.chula.ac.th; 4 Department of Pharmacology and Physiology, Faculty of Pharmaceutical Sciences, Chulalongkorn University, Bangkok 10330, Thailand; 5 Department of Biochemistry and Microbiology, Faculty of Pharmaceutical Sciences, Chulalongkorn University, Bangkok 10330, Thailand; 6 Natural Products and Nanoparticles Research Unit (NP2), Chulalongkorn University, Bangkok 10330, Thailand; kanokpol.a@alumni.chula.ac.th (K.A.); chaisak.c@pharm.chula.ac.th (C.C.); khanit.s@chula.ac.th (K.S.); supakarn.c@pharm.chula.ac.th (S.C.); 7 Department of Pharmacognosy and Pharmaceutical Botany, Faculty of Pharmaceutical Sciences, Chulalongkorn University, Bangkok 10330, Thailand

**Keywords:** 5-*O*-(*N*-Boc-l-alanine)-renieramycin T, *Xestospongia* sp., marine sponge, lung cancer, anti-cancer, cancer stem cells, apoptosis, Akt, c-Myc

## Abstract

Cancer stem cells (CSCs) drive aggressiveness and metastasis by utilizing stem cell-related signals. In this study, 5-*O*-(*N*-Boc-l-alanine)-renieramycin T (OBA-RT) was demonstrated to suppress CSC signals and induce apoptosis. OBA-RT exerted cytotoxic effects with a half-maximal inhibitory concentration of approximately 7 µM and mediated apoptosis as detected by annexin V/propidium iodide using flow cytometry and nuclear staining assays. Mechanistically, OBA-RT exerted dual roles, activating p53-dependent apoptosis and concomitantly suppressing CSC signals. A p53-dependent pathway was indicated by the induction of p53 and the depletion of anti-apoptotic Myeloid leukemia 1 (Mcl-1) and B-cell lymphoma 2 (Bcl-2) proteins. Cleaved poly (ADP-ribose) polymerase (Cleaved-PARP) was detected in OBA-RT-treated cells. Interestingly, OBA-RT exerted strong CSC-suppressing activity, reducing the ability to form tumor spheroids. In addition, OBA-RT could induce apoptosis in CSC-rich populations and tumor spheroid collapse. CSC markers, including prominin-1 (CD133), Octamer-binding transcription factor 4 (Oct4), and Nanog Homeobox (Nanog), were notably decreased after OBA-RT treatment. Upstream CSCs regulating active Akt and c-Myc were significantly decreased; indicating that Akt may be a potential target of action. Computational molecular modeling revealed a high-affinity interaction between OBA-RT and an Akt molecule. This study has revealed a novel CSC inhibitory effect of OBA-RT via Akt inhibition, which may improve cancer therapy.

## 1. Introduction

Lung cancer is an important human cancer. At present, several strategies are used for lung cancer treatment, including surgery, chemotherapy, radiotherapy, and targeted therapy; however, drug resistance and the spread of the cells to form metastases frequently result in poor prognosis and treatment failure. Advances in molecular and clinical research have highlighted the role of a cancer cell population, namely cancer stem cells (CSCs), and the concept of CSCs has dramatically altered the understanding view of cancer cell biology, pathogenesis, and the clinical response [1]. Therefore, the current drug discovery theme has focused on the undifferentiated cancer cell population, as the available therapy primarily eradicates the non-CSC population in the tumor, thereby sparing drug-resistant CSCs [2]. High tumorigenic potentials augment cellular survival and drug-resistant mechanisms, and the metastatic abilities of lung CSCs depend on the specific stem cell-related signaling pathways [3]. 

Myc is a family of proto-oncoproteins that regulate cell growth, survival, and proliferation [4]. In lung cancer, c-Myc is recognized as a key factor facilitating cell growth, drug resistance, and dissemination. In addition, its dominant role in controlling CSC properties supports the concept that targeting c-Myc could be a potential method for lung cancer therapy [5]. A number of studies and observations show the co-incidence of c-Myc and activated PI3K/Akt in transformed cells. Moreover, the PI3K/Akt/c-Myc signaling axis could promote CSC properties in cancers [6]. 

The deregulation of Akt is associated with several features of cancers, and Akt-targeting compounds can improve cancer therapies. In addition, a number of Akt inhibitors have been investigated for lung cancer treatment [7]. Natural tetrahydroisoquinoline of marine origin and their analogs, such as ecteinascidins from tunicates, exhibit potent cytotoxicity against several types of cancer cells, and they have been approved for clinical use in the treatment of cancers, including advanced soft-tissue sarcoma and ovarian cancer in the case of ecteinascidin 743 (trabectedin) [8] and metastatic small cell lung cancer in the case of the semisynthetic analog, namely lurbinectedin [9]. 

However, the mechanism of action of ecteinascidins is not fully understood. Ecteinascidins can exert anticancer activities via binding with DNA and DNA-binding proteins and mediating cell apoptosis [10]. Ecteinascidins target Akt as it can dramatically decrease phosphorylated Akt (s473-AKT or p-Akt) [11] and reduce the expression level of several anti-apoptotic proteins, such as Bcl-2 and Mcl-1 [12]. A recent pre-clinical study in the xenograft mice model of uterine cervical cancer revealed that lurbinectedin effectively eliminates CSCs [9]. Renieramycins, which are bis(tetrahydroisoquinoline)quinone alkaloids found in sea sponges and nudibranchs, are members of the same soframycin family as tris(tetrahydroisoquinoline) ecteinascidins [13] and have also demonstrated potent anticancer activities [14], particularly renieramycin M (RM), which is the major bis(tetrahydroisoquinolinequinone) constituent isolated from potassium cyanide-pretreated *Xestospongia* sp. collected in Thailand [15] and the Philippines [16]. RM can sensitize resistance to anoikis via decreasing cellular levels of survival and apoptotic proteins (including p-Akt, p-ERK, Bcl-2, and Mcl-1) [15] and attenuate CSC-like phenotypes [17] in H460 cells. Similar to derivatizations of ecteinascidins [18], late-stage modifications on either the A- or E-ring quinone of RM have been found to alter the mode of action and cellular targeting pattern, which can lead to enhanced selectivity and activity. A series of 5-*O*-Boc-amino ester derivatives of RM is synthesized and successfully used in a structural cytotoxicity relationship study [19], indicating that Boc-protected amino acid moieties serve as empirical groups in the introduction of additional compound–target intermolecular interaction networks and modifying their physicochemical properties. The cinnamoyl ester derivative of RM has superior cytotoxicity compared with the parent compound [20], and it can suppress CSCs potentially by inhibiting Akt [21]. Renieramycin T (RT), a hybrid renieramycin–ecteinascidin analog derived from RM with a methylhydroxybenzodioxole unit resembling ecteinascidin [22], could promote p53-dependent apoptosis via near-to-complete depletion of Mcl-1 and partly decrease the cellular level of Bcl-2, whereas RT did not affect Akt [23]. A trabectedin-mimic derivative of RT, 5-*O*-acetyl-renieramycin T (*O*-acetyl RT), could significantly deplete Akt and reverse CSC-associated cisplatin resistance in non-small-cell lung carcinoma (NSCLC) [24]. Protections at the phenolic alcohol at C-5 can enhance the cytotoxicity of RT derivatives [25]. Based on previous developments, we synthesized the 5-*O*-(*N*-Boc-l-alanine)-renieramycin T (OBA-RT) from RM and investigated the induction of cancer cell apoptosis and the CSC-suppressing effect. Using molecular pharmacological and computational modeling approaches, we reported the potential CSC-targeting activities of this new compound, which could improve anticancer therapy.

## 2. Results

### 2.1. Semi-Synthesis of 5-O-(N-Boc-l-Alanine)-Renieramycin T (OBA-RT)

**Figure 1:**
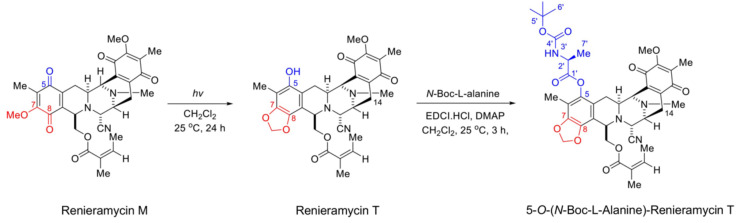
Semi-synthesis of 5-*O*-(*N*-Boc-l-alanine)-renieramycin T (OBA-RT). Photoconversion of renieramycin M to renieramycin T generated a phenolic alcohol at C-5 as a conjugation handle for Steglich esterification with *N*-Boc-l-alanine via EDCI/DMAP coupling, yielding OBA-RT.

OBA-RT was semi-synthesized from RM through a two-step reaction comprising the facile light-mediated conversion of RM into RT [26] and the *N*-Boc-l-alanine conjugation to RT by esterification (Figure 1). RM was irradiated with LED blue light and was subjected to photoredox transformation of methoxybenzoquinone into hydroxybenzo [1,3] dioxole, yielding RT with a 1,3-dioxole ring at C-7 and C-8, and a hydroxy group at C-5, which enabled the subsequent conjugation with *N*-Boc-l-alanine. The Steglich esterification of RT with *N*-Boc-l-alanine was performed using 1-ethyl-3-(3-dimethylaminopropyl)carbodiimide (EDCI)/4-dimethylaminopyridine (DMAP) as coupling reagents. In addition to high-resolution mass spectrometry and IR spectroscopy, the chromatographically purified OBA-RT was subjected to ^1^H and ^13^C NMR spectroscopy, which indicated the identity of the compound (see Supplementary Information for NMR spectra Figures S1–S5). The characteristic proton chemical shifts of the *N*-Boc-l-alanine motif included a broad doublet of a carbamate hydrogen (3′-NH) at 5.01 ppm, a triplet of the α-proton of alanine (2′-H) at 4.58 ppm, a doublet of methyl protons of alanine (7′-CH_3_) at 1.70 ppm, and the 9H-equivalent singlet of the tert-butyl group (6′-CH_3_) at 1.46 ppm. The characteristic pair of doublets of methylenedioxy protons at 5.97 ppm corresponded to the tetrahydroisoquinoline benzodioxole of renieramycin T. Regarding ^13^C-NMR spectrum, the *N*-Boc-l-alanine carbonyl of carbamate at C4′, ester carbonyl at C1′, α-carbon of alanine (C2′), methyl carbon of alanine (C7′) and methylenedioxy carbon peaks appeared at 158.3, 171.2, 49.3, 18.6, and 101.8 ppm, respectively. 

### 2.2. Cytotoxicity and Apoptosis-Inducing Effect of OBA-RT 

**Figure 2:**
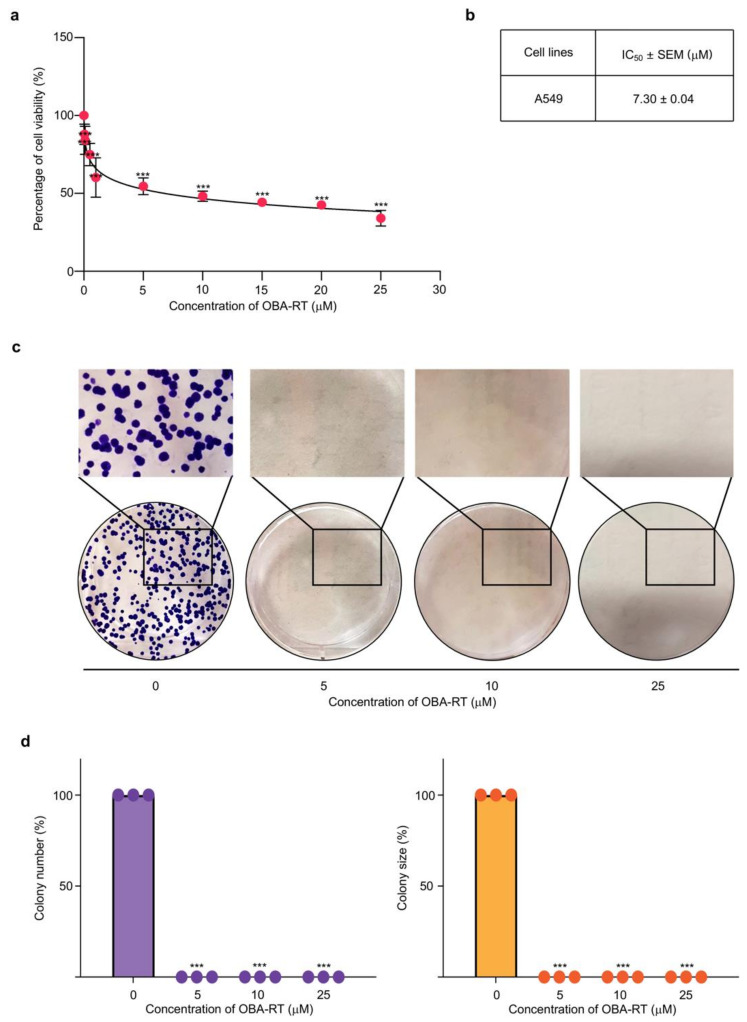
OBA-RT reduces the viability of non-small-cell lung cancer (NSCLC) cells. (**a**) A549 cells were treated with various concentrations of OBA-RT (0–25 µM) for 24 h. Cell viability was determined by MTT assay. (**b**) The half-maximal inhibitory concentration (IC_50_) at 24 h was calculated. (**c**,**d**) The effect of OBA-RT on colony formation of A549 cells was observed using a colony formation assay. Colonies were stained by crystal violet. Data are represented as the mean ± SEM (*n* = 3). *** *p* < 0.0001 compared with untreated control cells.

We determined the cytotoxic profile of OBA-RT in NSCLC A549 cells to elucidate the anticancer potential of OBA-RT. After treating the cells with various concentrations of OBA-RT (0–25 µM) for 24 h, cell viability was evaluated by using the 3-(4,5-dimethylthiazolyl-2)-2,5-diphenyltetrazolium bromide assay. The results showed that OBA-RT significantly reduced the viability of A549 cells (Figure 2a) with a half-maximal inhibitory concentration (IC_50_) value of 7.30 ± 0.07 µM (Figure 2b). The cytotoxic effects of OBA-RT were considered non-toxic at concentrations of ≤0.05 µM in A549 cells.

We confirmed the effect of OBA-RT in causing decreased cell survival by investigating the surviving cells after treatment by colony formation assay. Surviving A549 cells after treatment with OBA-RT (5, 10, and 25 µM) for 24 h were counted and seeded for the colony formation assay without further treatment. Crystal violet-stained colonies, showing the reproduction of a new cancer colony from a single cell, are shown in Figure 2c,d. The results showed that the resistant cells receiving OBA-RT at 5 to 25 µM could not form colonies (Figure 2c,d). 

### 2.3. OBA-RT Induced Apoptosis through p53 Activation 

**Figure 3:**
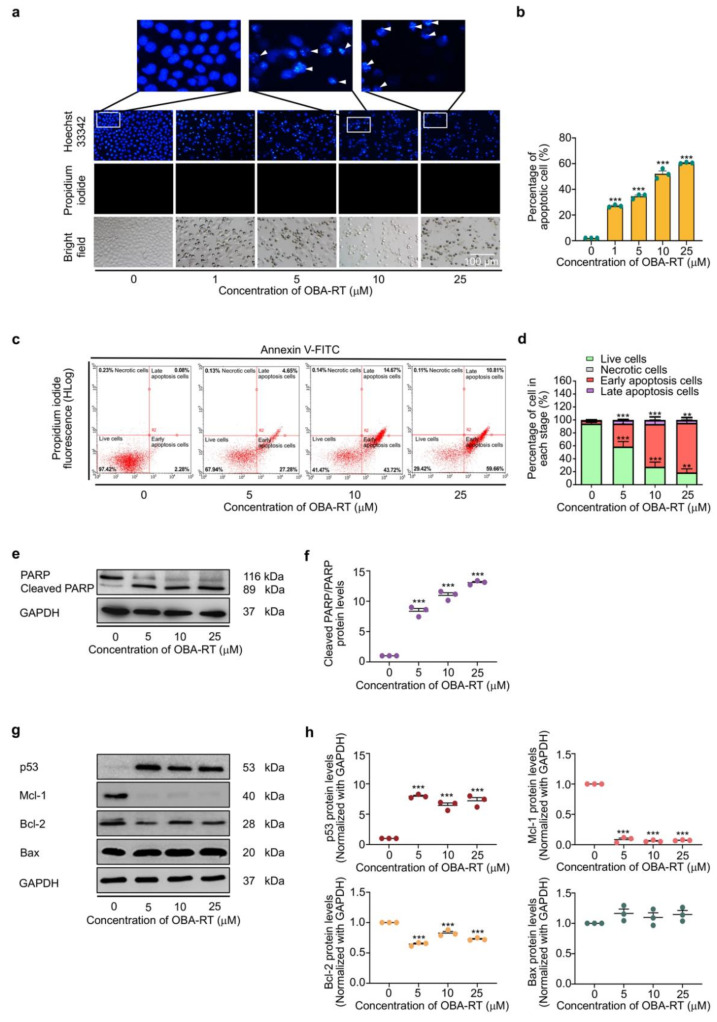
OBA-RT induces apoptosis in a p53-dependent manner. (**a**,**b**) The nuclei of A549 cells treated with OBA-RT were stained with Hoechst 33342/propidium iodide (PI) and calculated as a percentage compared with untreated control cells. The fragmented nuclei in apoptotic cells were indicted by arrowheads. (**c**) Apoptotic and necrotic cell death was determined using the annexin V/PI staining assay. (**d**) Percentages of cells at each stage were calculated. (**e**) OBA-RT at a concentration of 0-25 µM for 24 h induces cleavage of PARP, as examined by Western blot analysis. (**f**) Relative protein levels were quantified by densitometry. (**g**) The expression levels of apoptosis-associated proteins Bcl-2, Mcl-1, Bax, and p53 in A549 cells treated with OBA-RT (0–25 µM) for 24 h were examined by Western blot analysis. To confirm equal loading of the protein samples, the blots were reprobed with the GAPDH antibody. (**h**) Relative protein levels were quantified by densitometry. Data are presented as mean ± SEM (*n* = 3). *** *p* < 0.0001 compared with untreated control cells.

In determining the mode of cell death induced by OBA-RT, A549 cells were treated with OBA-RT (0–25 µM) for 24 h, and the apoptosis and necrosis cells were quantified using the Hoechst 33342/propidium iodide (PI) double staining assay. Hoechst 33342 staining was used to evaluate the nuclear morphology of apoptotic cells, showing condensed or fragmented nuclei, whereas PI stains the nucleus of necrotic cells. The results indicate that OBA-RT could increase apoptosis in a dose-dependent manner, whereas necrotic cells were minimally detected in response to all treatments. Therefore, OBA-RT primarily induced apoptotic cell death in our experimental setting (Figure 3a,b). Other apoptotic cell features, including the presence of extracellular phosphatidylserine, were determined to confirm the apoptosis-inducing effect of OBA-RT. Flow cytometric analysis of annexin V/PI staining of the OBA-RT-treated cells showed that OBA-RT could increase the number of annexin V-positive apoptotic cells (Figure 3c). As shown in Figure 3d, the percentage of early apoptotic cells was 27.28%, 43.72%, and 59.66% in A549 cells treated with OBA-RT at concentrations of 5, 10, and 25 µM, respectively.

In addition, the specific apoptotic marker protein, namely poly (ADP-ribose) polymerase (PARP), and its cleaved form were detected in the treated cells. For mechanistic analysis, we monitored the alteration of apoptosis regulatory proteins, which belong to the Bcl-2 family and its upstream regulator p53 proteins. Protein determination was performed by Western blotting. Lung cancer cells were treated with OBA-RT (0–25 µM) for 24 h. Western blotting revealed that in response to OBA-RT treatment, the cleaved form of PARP was significantly increased compared with the untreated control cells, as shown in Figure 3e,f. For apoptosis induction, the major regulators of p53-dependent apoptosis, such as p53, anti-apoptotic proteins (Mcl-1 and Bcl-2), and pro-apoptotic proteins (Bax), were investigated in OBA-RT-treated cells. The results revealed that p53 was dramatically increased in response to compound treatment. Moreover, anti-apoptotic Bcl-2 and Mcl-1 were decreased, whereas pro-apoptotic Bax was found to be slightly altered (Figure 3g,h).

### 2.4. OBA-RT Suppresses CSC Spheroid Formation 

**Figure 4:**
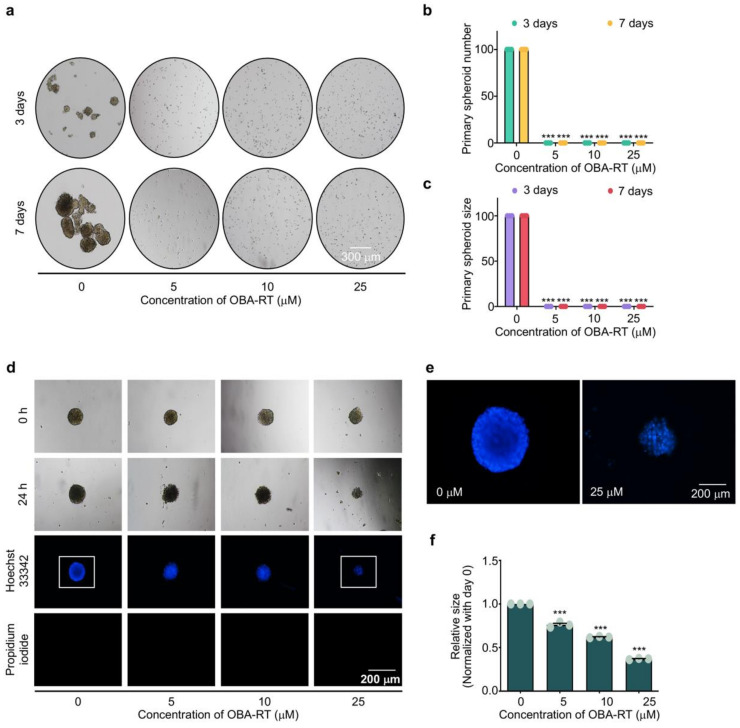
OBA-RT suppresses cancer stem cell (CSC) phenotypes in A549 human non-small-cell lung cancer cells. To assess the spheroid formation activity, (**a**) Cells were pre-treated with OBA-RT for 24 h and allowed to form primary spheroids for 7 days. The numbers (**b**) and sizes (**c**) of primary spheroids were calculated. (**d**) To further confirm the CSC-killing activity of OBA-RT, the CSC-rich populations in 3D culture were established by forming primary spheroids for 7 days. The primary spheroids were suspended into single cells to form CSC-rich spheroids for 14 days in ultralow-attachment 96-well plates. The CSC-rich spheroids were then treated with OBA-RT at concentrations of 0–25 µM for 24 h. (**e**) The apoptotic cells were determined by Hoechst 33342 staining. (**f**) Relative size of CSC spheroids was quantified. Data are presented as mean ± SEM (*n* = 3). *** *p* < 0.0001 compared with untreated control cells.

CSCs have become an important target for the determination of novel anticancer drugs. The ability of cancer cells to form tumor spheroids has been referred to as augmented CSC potential. Next, we tested whether OBA-RT possessed CSC-suppressing activity. A549 cells were treated with OBA-RT at concentrations of 0-25 µM for 24 h, and the cells were subjected to a spheroid formation assay. The results showed that the cells treated with OBA-RT (5–25 µM) exhibited a reduced ability to form tumor spheroids in a concentration-dependent manner (Figure 4a–c). To further confirm the CSC-killing population, the apoptotic induction of OBA-RT in the CSC population of A549 cells was elucidated. A CSC-rich population was established in the lung cancer cells. The CSC spheroids were seeded in 96-well plates by ultralow attachment at a density of one spheroid per well. The spheroids were treated with OBA-RT (0–25 µM) for 24 h. In addition, the untreated spheroids exhibited normal survival features, and the OBA-RT-treated spheroids detached and dissociated (Figure 4d). Hoechst 33342 staining of the treated spheroids further revealed the apoptotic character of DNA fragmentation and/or DNA condensation in the OBA-RT-treated spheroids (Figure 4d–f). Collectively, OBA-RT possessed anti-CSC phenotypes that could induce CSC apoptosis.

### 2.5. OBA-RT Suppresses CSC Signals in A549 Cells

**Figure 5:**
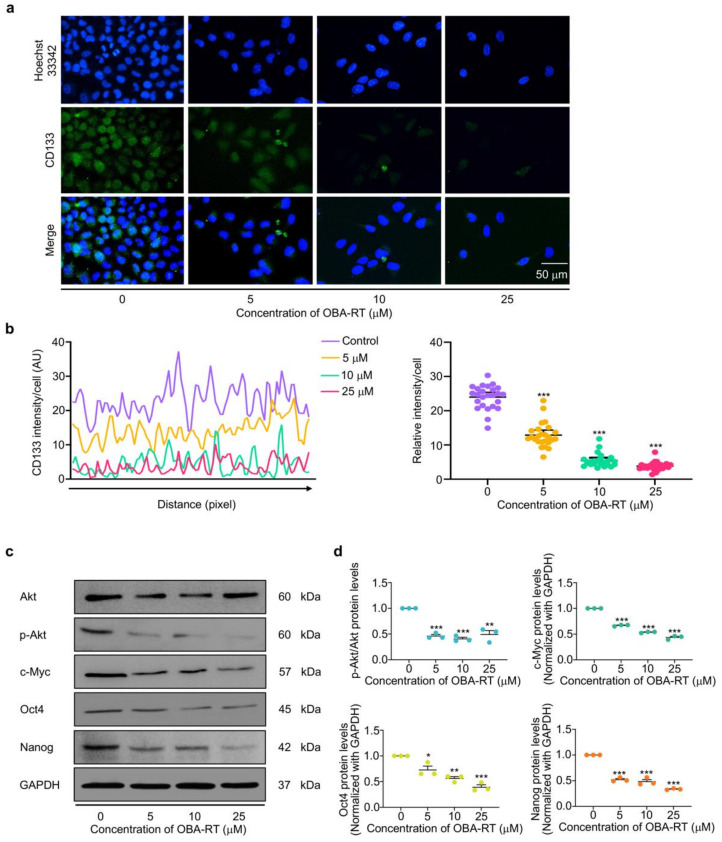
(**a**) OBA-RT inhibits Akt and suppresses CSCs. A549 cells were treated with OBA-RT for 24 h. The cells were co-stained with CD133 antibodies and Hoechst 33342. The expression of CD133 was examined by immunofluorescence (IF). (**b**) The fluorescence intensity was analyzed by ImageJ software. (**c**) The expression of activated Akt (p-Akt), total Akt, and the expression levels of stemness-related proteins Oct4, Nanog, and c-Myc in A549 cells treated with OBA-RT (0–25 uM) for 24 h were examined by Western blot analysis. To confirm equal loading of the protein samples, the blots were reprobed with the GAPDH antibody. (**d**) Relative protein levels were quantified by densitometry. Data are presented as mean ± SEM (*n* = 3). * 0.01 ≤ *p* < 0.05, ** *p* < 0.01, *** *p* < 0.0001 compared with untreated control cells.

We determined CD133, a well-known CSC marker in response to compound treatment, to confirm the CSC-suppressing effect of OBA-RT. The cells were similarly treated with 0–25 µM OBA-RT for 24 h. The level of CD133 was then analyzed by immunofluorescence detected by a specific CD133 antibody. Figure 5a,b show that CD133 fluorescence intensity at concentrations of 5–25 µM significantly decreased when compared with the non-treatment control.

Inhibiting CSC-maintaining cellular signals is a potential way to reduce and improve clinical outcome in CSC-driven cancers, including lung cancer. The stemness properties of cancer are regulated by several pathways, and the Akt pathway can regulate pluripotent transcription factors, namely Nanog and Oct4. Considering that OBA-RT could suppress the CSC phenotypes in lung cancer cells, we further tested whether this compound could effectively inhibit the CSC upstream signals via Akt inhibition and deplete the transcription factors of stem cells. The A549 cells were treated with various concentrations of OBA-RT (0–25 µM) for 24 h. In addition, CSC transcription factors, namely Oct4 and Nanog, and CSC regulatory proteins, namely Akt, p-Akt, and c-Myc proteins, were analyzed by Western blotting analysis. The results revealed that Nanog, Oct4, and c-Myc were significantly decreased after OBA-RT treatment at concentrations of 5 and 25 µM. Akt signaling was highlighted as a therapeutic target for CSC-driven and malignant cancers; thus, the protein expression ratio of phosphorylated Akt/Akt was evaluated. After treatment of OBA-RT (5–25 µM) for 24 h, the p-Akt/Akt ratio was dramatically diminished when compared with the non-treatment control (Figure 5c,d).

### 2.6. Molecular Docking Simulations Indicated the OBA-RT Interactions with the Allosteric Pocket of Akt-1 Protein

**Figure 6:**
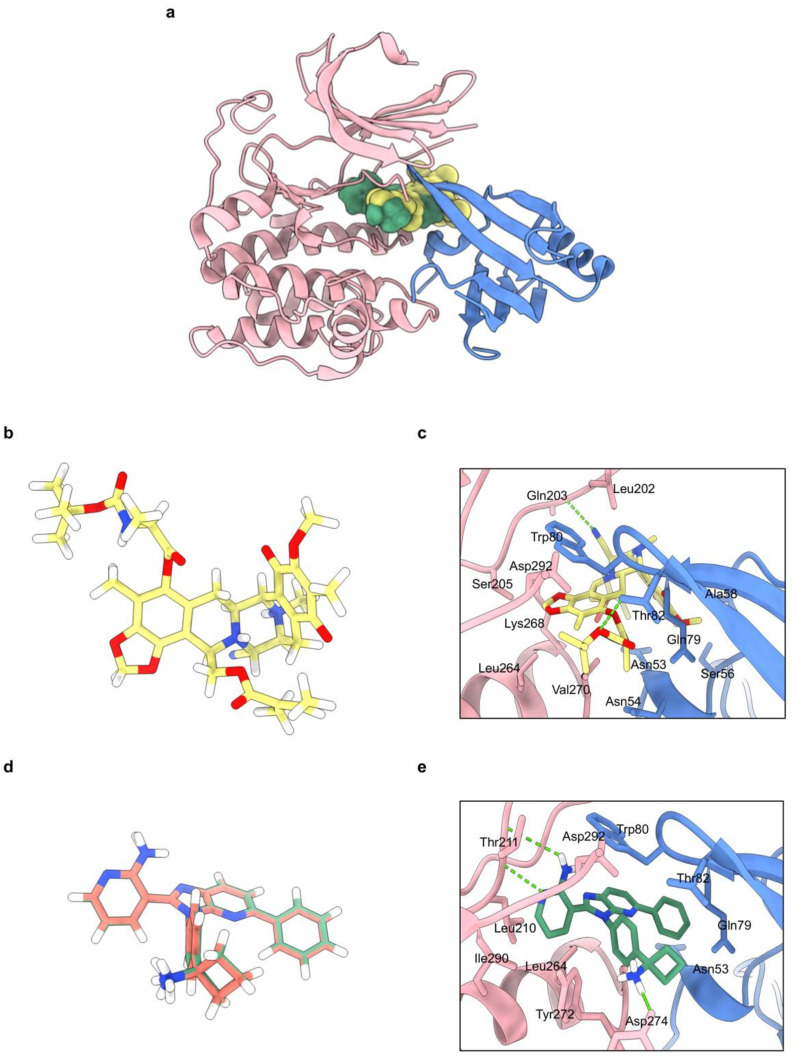
(**a**) Co-crystal structure of Akt-1 in complex with OBA-RT and miransertib (PDB code: 5KCV). The kinase domain is shown in pink, PH domain in blue, OBA-RT in yellow, and miransertib in green. (**b**) 3D chemical structure of OBA-RT. (**c**) The binding mode of OBA-RT to the allosteric pocket of Akt-1. One of two major hydrogen bonds was formed between the carbamate ether oxygen of Boc-l-alanine moiety and Thr82 while the butyl group was contributed to hydrophobic interactions with Leu264, Leu268, and Val270. (**d**) Redocking of miransertib in Akt-1 (PDB code: 5KCV); overlap of the co-crystal ligand miransertib (red) and redocking (green). (**e**) The binding mode of miransertib to the allosteric pocket of Akt-1. Hydrogen bonds are displayed as green dashed lines.

We performed a molecular docking simulation of OBA-RT with Akt (PDB code: 5KCV) to evaluate the possibility of a direct interaction between OBA-RT and Akt. In verifying the docking protocol, we redocked miransertib into its original binding site on Akt using Autodock Vina. The root mean square deviation (RMSD) of the redocked ligand was a small RMSD value (0.484 Å). The results (Figure 6d) indicated that the docking protocol was correct (RMSD < 2 Å) [27]. The binding energies of OBA-RT and co-crystal ligand miransertib have been reported in Table 1. OBA-RT could bind with Akt-1 with binding energy of −8.1 kcal/mol. As shown in Figure 6, OBA-RT forms two hydrogen bonds with Thr82 and Glu203 and forms hydrophobic interactions with Asn53, Asn54, Ser56, Ala58, Gln79, Trp80, Leu202, Ser205, Leu264, Lys268, Val270, and Asp292.

## 3. Discussion

We reported a facile and concise semi-synthesis of OBA-RT (Figure 1) from RM isolated from the blue sponge *Xestospongia* sp. using benzoquinone/naphthoquinone-type photoredox chemistry [28] and Steglich esterification. In addition, phototransformation might account for the abiotic formation of hydroxybenzodioxole in naturally occurring saframycin-type tetrahydroisoquinolinequinones as an alternative enzymatic oxidative cyclization for the biosynthesis [28]. This photosynthetic approach for C–H activation at C-5 would be useful for other tetrahydroisoquinolinequinones, such as jorunnamycins, although the extent of utility and compatibility with other substitutions require further investigation. Compared with the three-step hydrogenation/esterification/oxidation scheme [19], this synthesis strategy should be amenable for any 5-*O*-conjugation of RT and related compounds. For example, the synthesis of a series of amino acid RT conjugates to allow the study of the structure–activity relationship could be performed using this two-step scheme. Moreover, it could enable creative functionalization, such as antibody–drug conjugations, fluorescence dye ligation for microscopy, and activity-based or photoaffinity probes for a target engagement study based on proteomics.

In this study, our data indicated that OBA-RT has a cytotoxic effect on human A549 cells with an IC_50_ value of 7.30 ± 0.07 µM and displays molecular pharmacological properties in cancer cells similar to previously reported structurally related compounds (Figure 2a,b). Our study revealed that OBA-RT treatment could significantly inhibit cell viability (Figure 2c–d) by inducing apoptotic cell death (Figure 3a–d).

One important apoptotic pathway is the p53-dependent pathway. The tumor suppressor p53 protein plays an important role in regulating DNA repair, cell cycle arrest, and apoptotic cell death. In response to DNA damage, p53 was activated via Ataxia telangiectasia-mutated kinases [29]. The activation of p53 resulted in the alteration of the cellular balance of Bcl-2 family proteins, thereby increasing the pro-apoptotic members and decreasing the anti-apoptotic proteins. This alteration causes the release of the mitochondrial contents to the cytoplasm, and such contents motivate the function of caspases leading to apoptotic cell death. However, inducing apoptosis is not sufficient to eliminate cancer. In this research, the results show that OBA-RT has a mechanism of action similar to that of RT by inducing the p53-dependent signaling pathway and suppressing Mcl-1, which is an anti-apoptotic marker (Figure 3g,h). Interestingly, the protein analysis shows a predominant effect of the cellular protein levels of Mcl-1. Mcl-1 is an anti-apoptotic protein that has gained increasing interest in lung cancer cell biology because it is highly expressed in lung cancer [30]. Furthermore, Mcl-1 is important for the survival of lung cancer cells.

Particular populations of cancer cells, namely CSCs, have been reported as key driving factors for malignancy in several cancers. The conventional cancer therapy can only eliminate cancer cells and not CSCs. The CSCs can escape, resulting in the relapse of the disease in the future [2]. Indeed, different anti-CSC strategies have been assessed by inhibiting many intracellular signaling pathways, such as Wnt/TCF, signal transducer and activator of transcription 3, namely NF-κB and Akt. Akt signaling can be considered as a key regulator for cancers and CSC phenotypes. Notably, Akt signaling plays a critical role in regulating CSC maintenance and properties [31]. Previous studies have revealed that Akt is directly linked to the master pluripotency factor Oct4 [32] and regulating transcription factors Nanog and Sox2, and reversed therapy resistance [33]. A series of reports has shown that Akt inhibition may lead to CSC suppression. For example, Rhodes revealed that GSK690693 is a novel Akt kinase inhibitor that has recently entered phase I clinical trials. GSK690693 inhibited proliferation and induced apoptosis in a subset of tumor cells with potency consistent with the intracellular inhibition of Akt kinase activity that showed reductions in phosphorylated Akt substrates in vivo [34]. In 2019, Chantarawong reported that *O*-acetyl RT can suppress CSCs in lung cancer by depleting the AKT signal [24]. Interestingly, *O*-acetyl RT has a chemical structure similar to OBA-RT. Hongwiangchan also reported that CIN-RM suppressed CSCs by inhibiting the AKT signaling pathway, resulting in the downregulation of stem cell transcription factors, including Nanog, Oct4, and Sox2 [21]. Nanog and Oct4 are the key transcription factors that control self-renewal and the pluripotency of CSCs, and are prognostic biomarkers in lung CSCs under regulation of the Akt signaling pathway [35].

The inhibition of Akt at an essential binding site for protein activity is a powerful strategy. At present, several critical binding sites have been focused on. Consequently, allosteric Akt inhibitors have been highly emphasized because of their role in blocking the kinase activity of Akt and interfering a pleckstrin homology (PH)-domain membrane- mediated recruitment [36]. This inhibition prevents Akt kinase activation and phosphorylation. In this study, considering the allosteric mechanism of OBA-RT, we performed molecular docking simulations using the binding interaction pattern of OBA-RT with the allosteric pocket of Akt-1. The molecular docking result revealed that OBA-RT could bind with Akt-1 with a binding energy of −8.1 kcal/mol, which is suitable for a potential interaction with Akt. The binding interaction pattern of OBA-RT with the allosteric pocket of Akt-1 is illustrated in Figure 6. The allosteric pocket of Akt-1 was located between the kinase domain and N-terminal PH domain [37]. OBA-RT forms a hydrogen bond with Gln203 and hydrophobic interactions with Leu202, Ser205, Leu264, Lys268, Val270, and Asp292 in the kinase domain. Moreover, it forms a hydrogen bond with Thr82 and hydrophobic interactions with Asn53, Asn54, Ser56, Ala58, Gln79, and Trp80 in the PH domain. Trp80 has been reported as an important residue for the allosteric Akt-1 inhibitor [38]. The Boc- l-alanine extension contributed significantly to the overall affinity of OBA-RT to Akt; that is, the hydrogen bond formed between the carbamate ether oxygen and the hydroxyl group of Thr82 and the hydrophobic interaction formed between the terminal tert-butyl group and Leu264, Lys268, and Val270. In addition, OBA-RT showed a similar binding pattern compared with miransertib, an oral allosteric Akt-1 inhibitor, by hydrophobic interaction with Trp80. Thus, the analyses suggest that OBA-RT could interact with Akt-1 via an allosteric mechanism, which demonstrates the ability of OBA-RT to inhibit Akt-1, following the previous experimental results. This result could support the conclusion that OBA-RT could be a potential anticancer agent by targeting Akt activation through an allosteric mechanism. Based on our computational analysis, OBA-RT-resistant cell lines with Akt variants harboring mutations in a key residue predicted to directly bind to OBA-RT can be generated for experimental validation. In vitro biophysical analyses for the determination of binding parameters between Akt and OBA-RT, such as isothermal titration calorimetry and thermal shift assay, might be conducted to verify the target engagement. By functionalizing OBA-RT with biotin or a bio-orthogonal group to be used in pull-down assays, chemoproteomics could be used to identify the complete set of cellular targets beyond Akt.

The cellular target profiling provided in this work contributes to a new perspective on tetrahydroisoquinoline antitumor antibiotics and may inform further systematic medicinal chemistry development of compounds in this class with defined molecular pharmacology details for next-generation therapy for intractable cancers.

## 4. Materials and Methods

### 4.1. Reagents and Antibodies

Dulbecco’s Modified Eagle’s Medium (DMEM) medium, fetal bovine serum (FBS), penicillin/streptomycin, L-glutamine, phosphate-buffered saline (PBS), and trypsin-EDTA were obtained from Gibco (Grand Island, NY, USA). 3-(4,5-dimethylthiazol-2-yl)-2,5-Diphenyltetrazoliumbromide (MTT), dimethyl sulfoxide (DMSO), Hoechst 33342, propidium iodide (PI), and bovine serum albumin (BSA) were obtained from Sigma-Aldrich, Co. (St. Louis, MO, USA). The following primary antibodies, PARP (#9532), p53 (#9282), Mcl-1 (#94296), Bcl-2 (#4223), BAX (#5023), Akt (#9272), phosphorylated Akt (#4060), Nanog (#4903), Oct4 (#2840), c-Myc (#5605), and GADPH (#5174) were obtained from Cell Signaling Technology (Danvers, MA, USA). CD133 (#CA1217) was obtained from Cell Applications (San Diego, CA, USA). The respective secondary antibodies, anti-rabbit IgG (#7074) and anti-mouse (#7076), were obtained from Cell Signaling Technology (Danvers, MA, USA).

### 4.2. Semi-Synthesis of 5-O-(N-Boc-l-Alanine)-Renieramycin T (OBA-RT)

Renieramycin M was isolated from the Thai blue sponge *Xestospongia* sp. collected at Si-chang Island, in the Gulf of Thailand, with assistance from the Aquatic Recourses Research Institute, Chulalongkorn University, and permission from the Department of Fisheries, Ministry of Agriculture and Cooperatives, Thailand (0510.2/8234, Date 28 October 2019). The fresh blue sponge was mashed, pre-treated with potassium cyanide (10 mM) in phosphate buffer at pH 7, macerated in methanol, concentrated, extracted with ethyl acetate, and purified through silica gel column chromatography to obtain renieramycin M as an orange solid with an isolation yield of 0.02% *w*/*w* relative to the dry sponge [19].

Chemical reactions were carried out at room temperature (25 °C) using oven-dried glassware and magnetically stirred under an argon atmosphere using a balloon. The chemical reagents were purchased from Aldrich (Missouri, USA) and TCI (Tokyo, Japan). Anhydrous solvents were dried over 4Å molecular sieves. All reactions were monitored by thin-layer chromatography (TLC) performed using aluminum silica gel 60F254 (Merck, Darmstadt, Germany). Bands were identified by UV activity. Flash column chromatography was performed using 60 Å silica gel (230–400 mesh) as a stationary phase along with ethyl acetate and hexanes as a mobile phase. Regarding structure elucidations, infrared (IR) spectra were measured on a Perkin Frontier Fourier Transform Infrared Spectrometer. ^1^H and ^13^C nuclear magnetic resonance (NMR) spectra were obtained on a Bruker ADVANCE NEO 400 MHz NMR spectrometer, and deuterated chloroform (CDCl3) served as the internal standard for both ^1^H (7.27 ppm) and ^13^C (77.0 ppm) spectra. Accurate mass spectra were obtained using an Agilent 6540 UHD Q-TOF LC/MS spectrometer.

Renieramycin M (20 mg, 0.0347 mmol) was weighed into a round-bottom flask and dissolved in dry dichloromethane (30 mL). The orange reaction mixture was irradiated with 18 W fluorescent lamp [39]. The mixture was stirred vigorously at room temperature for 24 h under argon atmosphere. The reaction was monitored by TLC. Once all the starting material was consumed, 1-ethyl-3-(3-dimethylaminopropyl) carbodiimide (EDCI.HCl, 6.65 mg, 0.0347 mmol) and *N*, *N*-4-dimethylaminopyridine (DMAP, 4.24 mg, 0.0347 mmol) were added to the reaction. After stirring for 5 min, *N*-Boc-l-alanine was added (32.83 mg, 0.1735 mmol). The yellow reaction mixture was stirred at room temperature for 3 h under argon atmosphere. Next, the reaction was quenched by addition of water (5 mL). The organic layer was separated by separatory funnel and the aqueous layer was extracted with CH_2_Cl_2_ (10 mL, 3 times). The organic layers were combined, dried over anhydrous Na_2_SO_4_, filtered, and concentrated under reduced pressure. Purification by the silica gel flash chromatography eluting with hexanes:EtOAc (1:1) gave 6.1 mg (24%) of 5-*O*-(*N*-Boc-l-alanine)-renieramycin T as a brown amorphous solid. Chemical structure of 5-*O*-(*N*-Boc-l-alanine)-renieramycin T was elucidated by spectroscopic analysis as follows: IR (ATR) λ_max_ in cm^−1^: 3401.4, 2923.9, 2851.3, 1714.6,1654.0, 1456.7, 1410.0, 1376.8, 1305.7, 1233.5, 1150.6, 1093.7, 1043.8, 955.7, 769.9, 732.8, 557.1. ^1^H-NMR (CDCl_3_, 400 MHz) δ_H_ in ppm: 5.98 (1H, overlapped, 26-H), 5.97 (2H, dd, *J* = 22.4, 1.2, Hz, OCH_2_O), 5.01 (1H, br d, *J* = 7.2 Hz, 3′-NH), 4.58 (1H, t, *J* = 6.8 Hz, 2′-H), 4.53 (1H, dd, *J* = 11.6, 3.6 Hz, 22-H_a_), 4.02 (1H, dd, *J* = 11.6, 4.4, 22-H_b_), 4.16 (1H, overlapped, 1-H), 4.11 (1H, overlapped, 21-H). 3.97 (1H, overlapped, 11-H), 3.96 (3H, s, 17-OCH_3_), 3.36 (1H, d, *J* = 7.6, 13-H), 3.23 (1H, dt, *J* = 12.4, 2.8 Hz, 3-H), 2.73 (1H, dd, *J* = 20.8, 7.6 Hz, 14-H_α_), 2.55 (1H, m, 4-H_α_), 2.32 (1H, dd, *J* = 15.6, 7.6 Hz, 14-H_β_), 2.28 (3H, s, NCH_3_), 2.04 (3H, s, 6-CH_3_), 1.90 (3H, s, 16-CH_3_), 1.85 (3H, dq, *J* = 7.6, 1.2 Hz, 27-H_3_), 1.70 (3H, d, *J* = 7.2, 7′-H_3_), 1.61 (1H, overlapped, 4-H_β_), 1.66 (3H, s, 28-H_3_), 1.46 (9H, br s, 3 × 6′-CH_3_); ^13^C-NMR (CDCl_3_, 100 MHz) δ_C_ in ppm: 186.0 (C-15), 182.7 (C-18), 171.2 (C-1′), 167.0 (C-24), 158.3 (C-4′), 155.2 (C-17), 144.9 (C-7), 141.9 (C-20), 140.9 (C-8), 139.9 (C-5), 140.2 (C-26), 135.3 (C-19), 129.0 (C-16), 126.7 (C-25), 119.9 (C-6), 117.3 (21-CN), 112.3 (C-10), 112.1 (C-9), 101.8 (OCH_2_O), 80.1 (C-5′), 63.6 (C-22), 60.9 (17-OCH_3_), 59.2 (C-21), 56.3 (C-1), 55.5 (C-3), 54.8 (C-11), 54.7 (C-13), 49.3 (C-2′), 41.4 (NCH_3_), 28.3 (3 × 6′-CH_3_), 27.6 (C-14), 21.0 (C-4), 20.5 (28-CH_3_), 18.6 (7′-CH_3_), 15.9 (27-CH_3_), 9.5 (6-CH_3_), 8.6 (16-CH_3_). HR-ESI-MS *m*/*z* 747.3231 ([M+H]^+^, calculated for C_39_H_47_N_4_O_11_, 747.3236).

### 4.3. Preparation of the OBA-RT Stock Solution

OBA-RT was prepared by dissolving it in dimethyl sulfoxide (DMSO) solution and then stored at −20 °C. It was freshly diluted with medium to the desired concentrations before use. The final concentration of DMSO in solution was less than 0.5%, which caused no signs of cytotoxicity.

### 4.4. Cell Lines and Culture

Human non-small cell lung cancer (NSCLC) cell lines, A549 (ATCC^®^ CCL-185™, RRID: CVCL_0023) cells were obtained from the American Type Culture Collection (Manassas, VA, USA). A549 cells were cultured in DMEM. The medium was supplemented with 10% FBS, 2 mM L-glutamine, and 100 units/mL of each penicillin and streptomycin at 37 °C with 5% CO_2_ in a humidified incubator.

### 4.5. Cell Viability

A549 cells were cultured in 96-well plates at a density of 1 × 10^4^ cells/well and incubated overnight at environment 5% CO_2_. Cells were treated with OBA-RT at concentrations 0 to 25 µM for 24 h. After treatment, 100 µL of MTT reagent (0.4 mg/mL) was added to each well and incubated for 3 h. The formazan crystals were dissolved in DMSO and measured using a microplate reader (Anthros, Durham, NC, USA) at a wavelength of 570 nm.

### 4.6. Colony Formation Assay

The survival ability to colonize single cancer cells was investigated by colony formation assay. After treatment, cells were cultured into 6-well plates at density of 300 cells/well and incubated for 7 days. The cells were washed with 1X PBS, fixed with 4% paraformaldehyde (Sigma Chemical, St. Louis, MO, USA) for 30 min, and stained with 0.5% crystal violet solution. Cells were washed with 1 × PBS three times, and the number and sizes of colonies were counted.

### 4.7. Apoptotic Assay

A549 cells were seeded in 96-well plates at a density of 1 × 10^4^ cells/well and allowed to attach overnight. Cells were incubated with various concentrations of OBA-RT at 0 to 25 µM for 24 h, and then the cells were co-stained with 10 µM of Hoechst 33342 (Sigma, St. Louis, MO, USA) and propidium iodide (PI) (Sigma, St. Louis, MO, USA) for 30 min in darkness. Fluorescence microscopy (Olympus DP70, Melville, NY, USA) was performed to image the apoptotic cells.

In addition, Annexin V-FITC Apoptosis Kit (Thermo Fisher Scientific, Waltham, MA, USA) was assessed to investigate apoptotic and necrotics cells. A549 cells were seeded into 24-well plates at a density of 1.5 × 10^4^ cells/well and incubated overnight. Cells were treated with indicated concentrations of OBA-RT (0–25 µM) for 24 h, then harvested and suspended in the binding buffer followed by incubation with Annexin V and PI for 15 min in darkness. Apoptotic and necrotic cells were assessed by Guava easyCyte^TM^ flow cytometry (EMD Millipore, Hayward, CA, USA).

### 4.8. Spheroid Formation Assay

A549 cells were pre-treated with concentrations of OBA-RT (0–25 µM) for 24 h. The treated cells were seeded onto ultralow attachment plates at a density 2.5 × 10^3^ cells/well in DMEM containing 1% FBS (*v/v*) (Merck, DA, Germany) for 7 days to form spheroids. On days 3 and 7, the numbers and sizes of spheroids were determined using a phase-contrast microscopy (Nikon ECLIPSE Ts2, Tokyo, Japan).

### 4.9. CSC-Rich Population

The enrichment of the CSC subpopulation in cancer cells was successfully performed through the three-dimensional (3D) spheroid-formation assay. A549 cells were seeded onto 24-well ultralow attachment plates at approximately 2.5 × 10^3^ cells/well with serum-free medium to form primary spheroids for 7 days. After that, primary spheroids were resuspended into single cells and seeded onto 96-well ultralow attachment plates for 14 days to form CSC-rich spheroids. After 14 days, CSC-rich spheroids were treated with concentrations of OBA-RT (0–25 µM) for 24 h. After treatment, apoptotic cell death was analyzed with Hoechst 33342 and size of single spheroid was captured using phase-contrast microscopy (Nikon ECLIPSE Ts2, Tokyo, Japan).

### 4.10. Western Blot Analysis

A549 cells were seeded at a density of 4 × 10^5^ cells/well in 6-well plates overnight. Cells were treated with OBA-RT (0–25 µM) for 24 h. Then, cells were washed with cold 1X PBS and incubated in RIPA buffer, 1% Triton X-100, 100 mM PMSF, and a protease inhibitor for 30 min on ice. Protein concentrations were quantified using BCA protein assay kit from Pierce Biotechnology (Rockford, IL, USA). Cell lysates were separated by sodium dodecyl sulfate polyacrylamide gel electrophoresis (SDS-PAGE) and transferred to polyvinylidene difluoride (PVDF) (Bio-Rad Laboratories Inc., Hercules, CA, USA). The membrane was blocked with 5% (*w/v*) non-fat dry milk power (Merck, Darmstadt, Germany) at room temperature for 2 h and each membrane was incubated with the specific primary antibodies for overnight at 4 °C, as well as incubated with horseradish peroxidase (HRP)-conjugated secondary antibodies (Cell Signaling, Danvers, MA, USA) for 2 h at room temperature. The protein expression was observed using chemiluminescence (Supersignal West Pico; Pierce, Rockford, IL, USA) and quantified using ImageJ software (NIH, Bethesda, MD, USA).

### 4.11. Immunofluorescence Assay

A549 cells were seeded into 96-well plates at a density 1 × 10^4^ cells/well and incubated overnight. After treatment with OBA-RT for 24 h, the cells were fixed with 4% paraformaldehyde for 30 min, permeabilized with 0.5% Triton-X for 5 min, and blocked with 4% BSA for 1 h at room temperature. The cells were incubated with an anti-CD133 antibody overnight at 4 °C and then incubated with secondary antibody for 1 h, stained with Hoechst 33342 (Sigma, St. Louis, MO, USA) for 30 min at room temperature in darkness, and mounted using 50% glycerol (Merck, Darmstadt, Germany). Confocal images were assessed under fluorescence microscope (Nikon ECLIPSE Ts2, Tokyo, Japan) and analyzed by ImageJ software.

### 4.12. Molecular Docking

The crystal structure of miransertib (ARQ092) in complex with the Akt-1 [40] was retrieved from the Research Collaboratory for Structural Bioinformatics Protein Data Bank (PDB code: 5KCV) [41]. All water molecules and co-crystal ligand were deleted with the UCSF ChimeraX [42]. AutoDockTools version 1.5.7 [43] was used to repair missing atoms and add polar hydrogen atoms. The structure of OBA-RT was constructed utilizing MarvinSketch and optimized with the Gaussian 09 program [44] using density functional theory (DFT) with a B3LYP/6-31G (d,p) basis set. Autodock Vina [45] was performed to investigate an interaction between OBA-RT and the allosteric pocket of Akt-1 using default parameters. A grid box was set with the center of the co-crystal ligand (PDB code: 5KCV). The grid size was set to 20 × 20 × 20 Å with a spacing of 1 Å. Furthermore, visualization of binding interaction patterns was carried out by UCSF ChimeraX.

### 4.13. Statistical Analysis

All results were compared and expressed as mean ± standard error of the mean (SEM) from at least triplicate independent experiments. Statistical analyses were evaluated using analysis of variance (ANOVA) followed by Tukey’s HSD post hoc test. The statistic was calculated by using SPSS version 28 (IBM Corp., Armonk, NY, USA). Statistically significant differences were indicated by * *p*-values less than 0.05. GraphPad Prism 9 was used for creating graphs in all experiments (GraphPad Software, San Diego, CA, USA).

## 5. Conclusions

**Figure 7:**
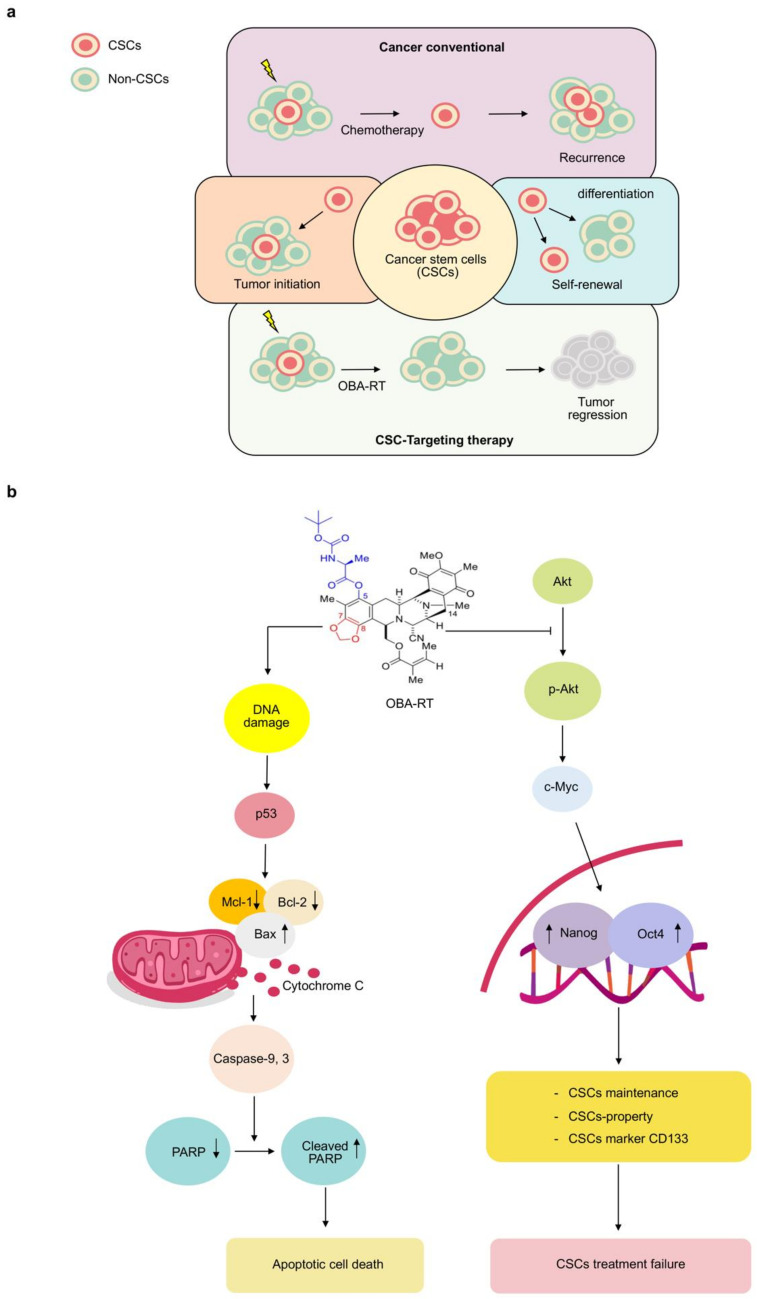
The proposed regulatory pathway of OBA-RT in inhibition of CSC and induction of apoptosis. (**a**) CSCs drive cancer initiation, progression, and therapeutic failure due to their abilities to initiate cancer, induce self-renewal and tumorigenicity, and augment pluripotent signals. CSCs are known to be highly resistant to chemotherapy and cause cancer relapse. Specific treatment to CSCs may induce cancer collapse and prevent the relapse of the disease. (**b**) Akt signaling pathways are critical for CSC properties and apoptotic cell death leading to cancer aggressiveness. OBA-RT could inhibit Akt function, resulting in the induction of apoptosis and cancer stem cell suppression activity in lung cancer cells.

These results provide novel and significant data on the new derivative of RT (OBA-RT) suggesting it can be considered as a potential therapy for lung CSCs. The compound has a potent apoptotic and CSC-suppressing activity in lung cancer cells (Figure 7a,b). In addition, the OBA-RT molecule could exert allosteric inhibition of the Akt protein. As Akt is critical for cancer cell survival and stemness phenotypes, our results might be used in demonstrating OBA-RT as a potential therapy for CSC and Akt-driven cancers.

## Figures and Tables

**Table 1:** Binding energy in kcal/mol of OBA-RT compared to co-crystal ligand miransertib.

Compounds	Binding Energy (kcal/mol)	Hydrogen Bond Interactions	Hydrophobic Interactions
Miransertib (Co-crystal ligand)	−12.8	Thr211, Tyr272	Asn53, Gln79, Trp80, Thr82, Leu210, Leu264, Val270, Tyr272, Asp274, Ile290
OBA-RT	−8.1	Thr82, Glu203	Asn53, Asn54, Ser56, Ala58, Gln79, Trp80, Leu202, Ser205, Leu264, Lys268, Val270, Asp292

## Data Availability

Data is contained within the article.

## Supplementary Materials

The following are available online at https://www.mdpi.com/article/10.3390/md20040235/s1, Figure S1: ^1^H-NMR (400 MHz) spectrum of 5-*O*-(*N*-Boc-l-alanine)-renieramycin T in CDCl_3_; Figure S2: ^13^C-NMR (400 MHz) spectrum of 5-*O*-(*N*-Boc-l-alanine)-renieramycin T in CDCl_3_; Figure S3: COSY (400 MHz) spectrum of 5-*O*-(*N*-Boc-l-alanine)-renieramycin T in CDCl_3_; Figure S4: HSQC (400 MHz) spectrum of 5-O-(*N*-Boc-l-alanine)-renieramycin T in CDCl_3_; Figure S5: HMBC (400 MHz) spectrum of 5-*O*-(*N*-Boc-l-alanine)-renieramycin T in CDCl_3_.
