# Supplementary material for: 5-O-(N-Boc-l-Alanine)-Renieramycin T Induces Cancer Stem Cell Apoptosis via Targeting Akt Signaling
Source: Mar Drugs. 2022 Mar 29;20(4):235. doi: 10.3390/md20040235 (PMC9029977; doi:10.3390/md20040235)
Supplement: Supplementary file 1 [file marinedrugs-20-00235-s001.zip › marinedrugs-1608166-supplementary.pdf]

Supporting Information

5-*O*-(*N*-Boc-L-Alanine)-Renieramycin T Induces Cancer Stem Cell Apoptosis via Targeting Akt signalling

Darinthip Suksamai [1,2], Satapat Racha[3], Nicharat Sriratanasak [1,4], Chatchai Chaotham[1,5], Kanokpol Aphicho[6], Aye Chan Khine Lin[2,6], Chaisak Chansriniyom[6,7], Khanit Suwanborirux[6,7], Supakarn Chamni [6,7] and Pithi Chanvorachote [1,4*]

1 Center of Excellence in Cancer Cell and Molecular Biology, Faculty of Pharmaceutical Sciences, Chulalongkorn University, Bangkok 10330, Thailand; pithi.c@chula.ac.th (P.C.); darintip.ss@gmail.com (D.S.); nicharat.sri@outlook.com (N.S.); cchoatham@gmail.com (C.C.)
2 Graduate Program of Pharmaceutical Science and Technology, Faculty of Pharmaceutical Science, Chulalongkorn University, Bangkok, Thailand; darintip.ss@gmail.com (D.S.); ayechan23792@gmail.com (A.L.)
3 Interdisciplinary Program in Pharmacology, Graduate School, Chulalongkorn University, Bangkok, Thailand; satapatto@gmail.com (S.R.)
4 Department of Pharmacology and Physiology, Faculty of Pharmaceutical Sciences, Chulalongkorn University, Bangkok 10330, Thailand; pithi.c@chula.ac.th (P.C.); nicharat.sri@outlook.com (N.S.)
5 Department of Biochemistry and Microbiology, Faculty of Pharmaceutical Sciences, Chulalongkorn University, 10330 Bangkok, Thailand; cchoatham@gmail.com (C.C.)
6 Natural Products and Nanoparticles Research Unit (NP²), Chulalongkorn University, Bangkok 10330, Thailand; kanokpol.ap@gmail.com (K.A.); ayechan23792@gmail.com (A.C.K.L.); chaisak.c@pharm.chula.ac.th (C.C.); khanit.s@chula.ac.th (K.S.); supakarn.c@pharm.chula.ac.th (S.C.)
7 Department of Pharmacognosy and Pharmaceutical Botany, Faculty of Pharmaceutical Sciences, Chulalongkorn University, Bangkok 10330, Thailand; chaisak.c@pharm.chula.ac.th (C.C.); khanit.s@chula.ac.th (K.S.); supakarn.c@pharm.chula.ac.th (S.C.)
* Correspondence: pithi.c@chula.ac.th; Tel.: +662-218-8344

Content **Page**

Figure S1 ^{1}H-NMR (400 MHz) spectrum of 5-*O*-(*N*-Boc-*L*-alanine)-renieramycin T in $CDCl_3$ S2
Figure S2 ^{13}C-NMR (400 MHz) spectrum of 5-*O*-(*N*-Boc-*L*-alanine)-renieramycin T in $CDCl_3$ S3
Figure S3 COSY (400 MHz) spectrum of 5-*O*-(*N*-Boc-*L*-alanine)-renieramycin T in $CDCl_3$ S4
Figure S4 HSQC (400 MHz) spectrum of 5-*O*-(*N*-Boc-*L*-alanine)-renieramycin T in $CDCl_3$ S5
Figure S5 HMBC (400 MHz) spectrum of 5-*O*-(*N*-Boc-*L*-alanine)-renieramycin T in $CDCl_3$ S6

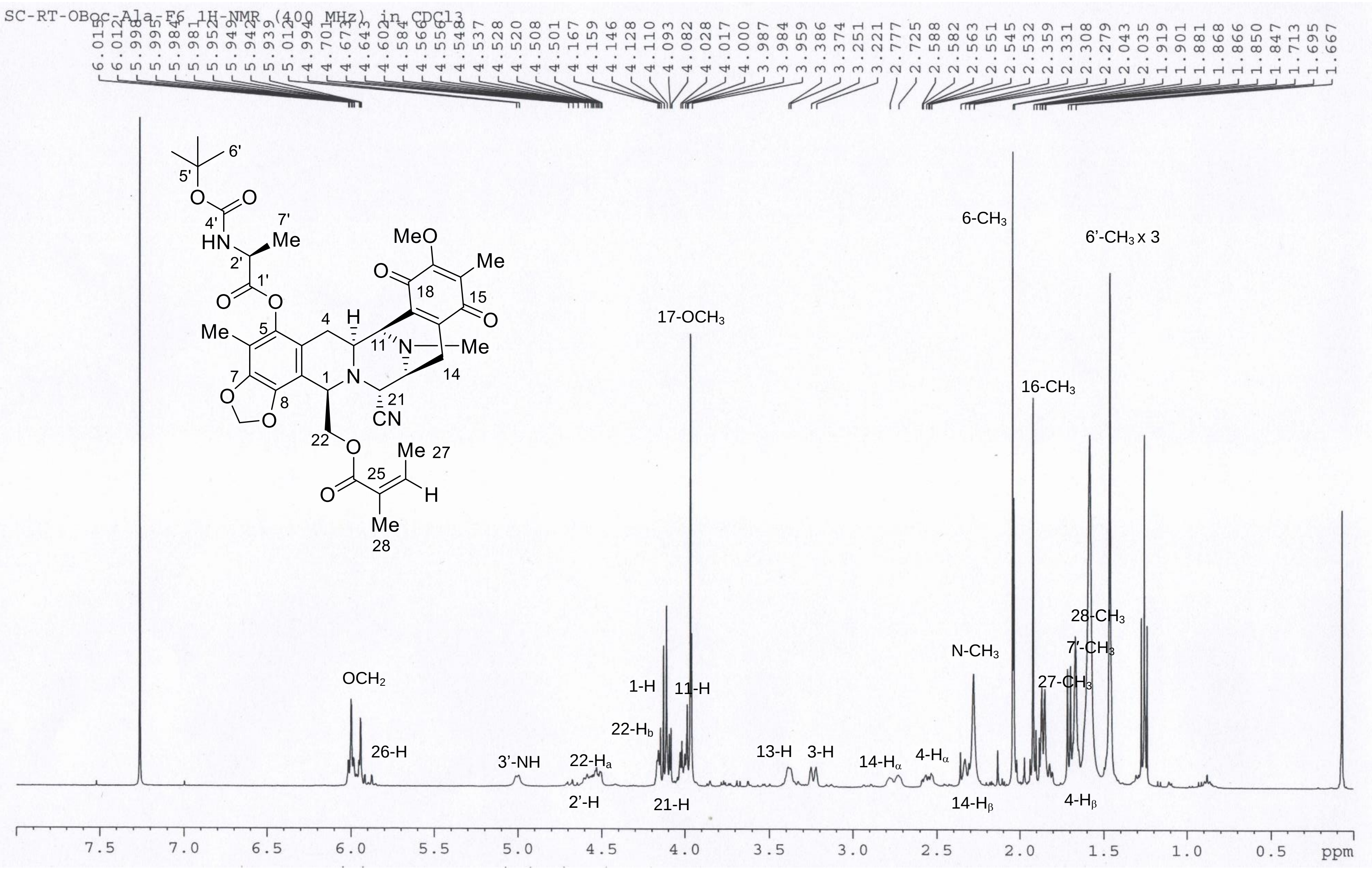

Figure S1. ^{1}H NMR (400 MHz) spectrum of 5-*O*-(*N*-Boc-L-alanine)-renieramycin T in $CDCl_3$

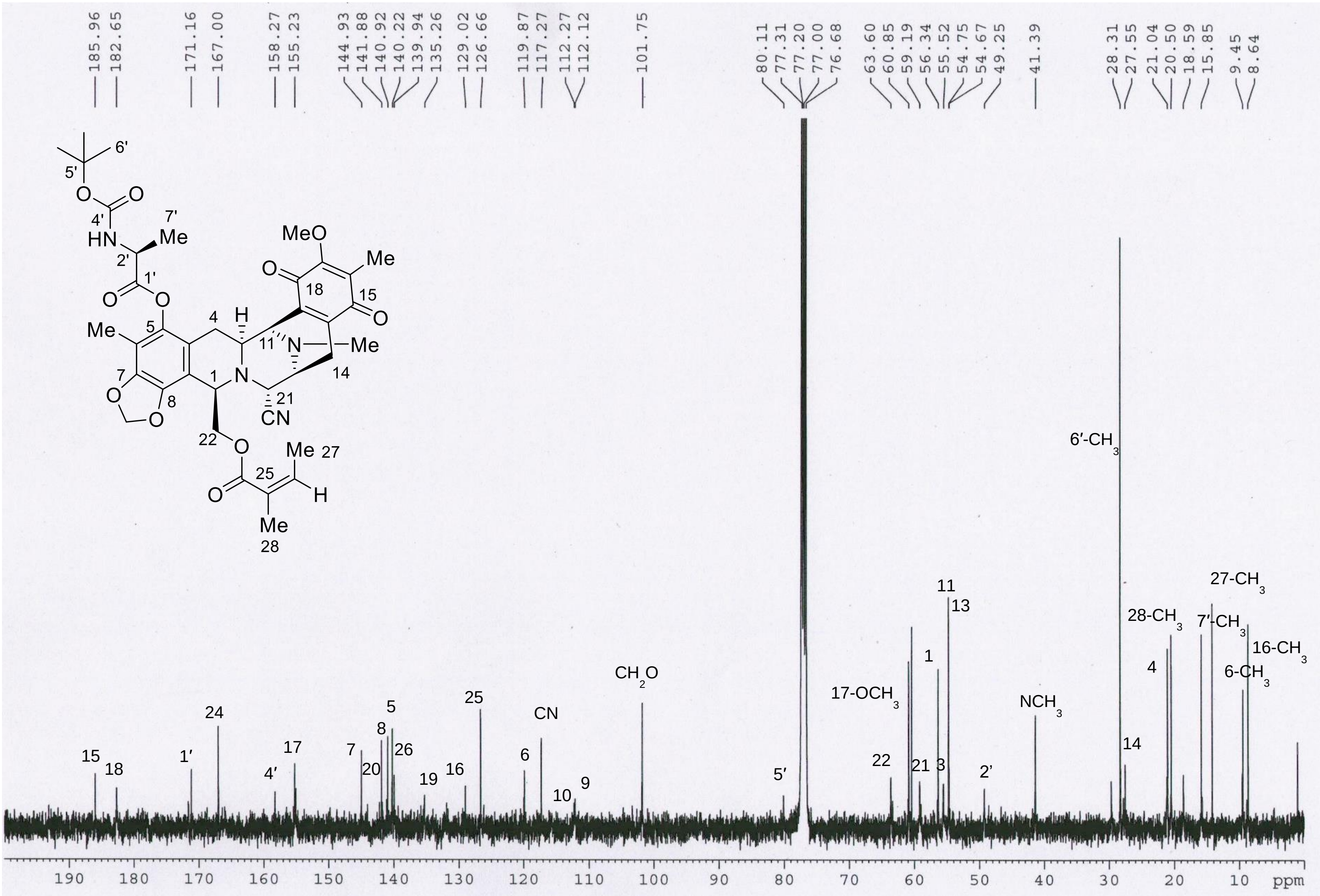

Figure S2. ^{13}C-NMR (400 MHz) spectrum of 5-*O*-(*N*-Boc-L-alanine)-renieramycin T in $CDCl_3$

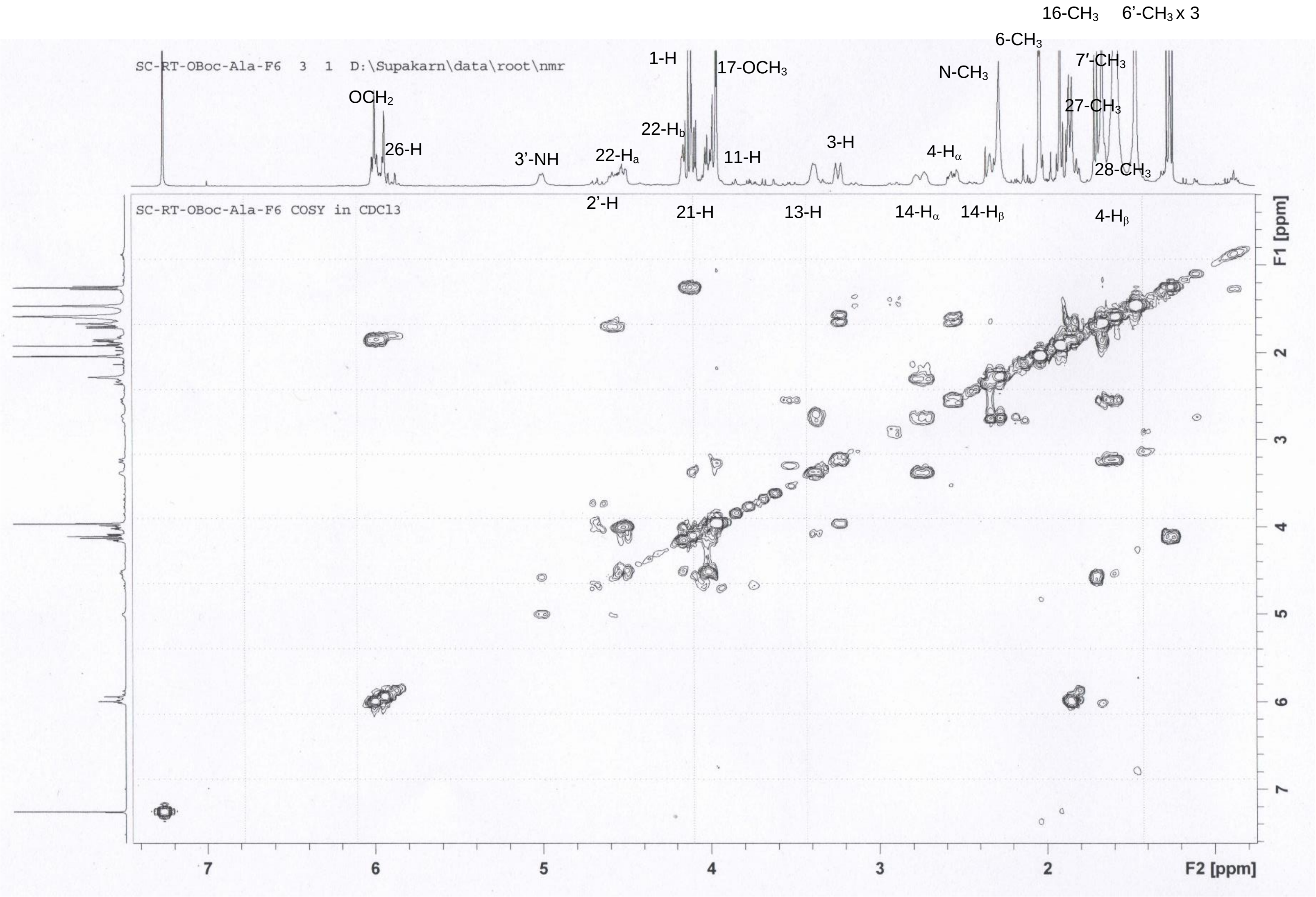

Figure S3. COSY (400 MHz) spectrum of 5-*O*-(*N*-Boc-L-alanine)-renieramycin T in $CDCl_3$

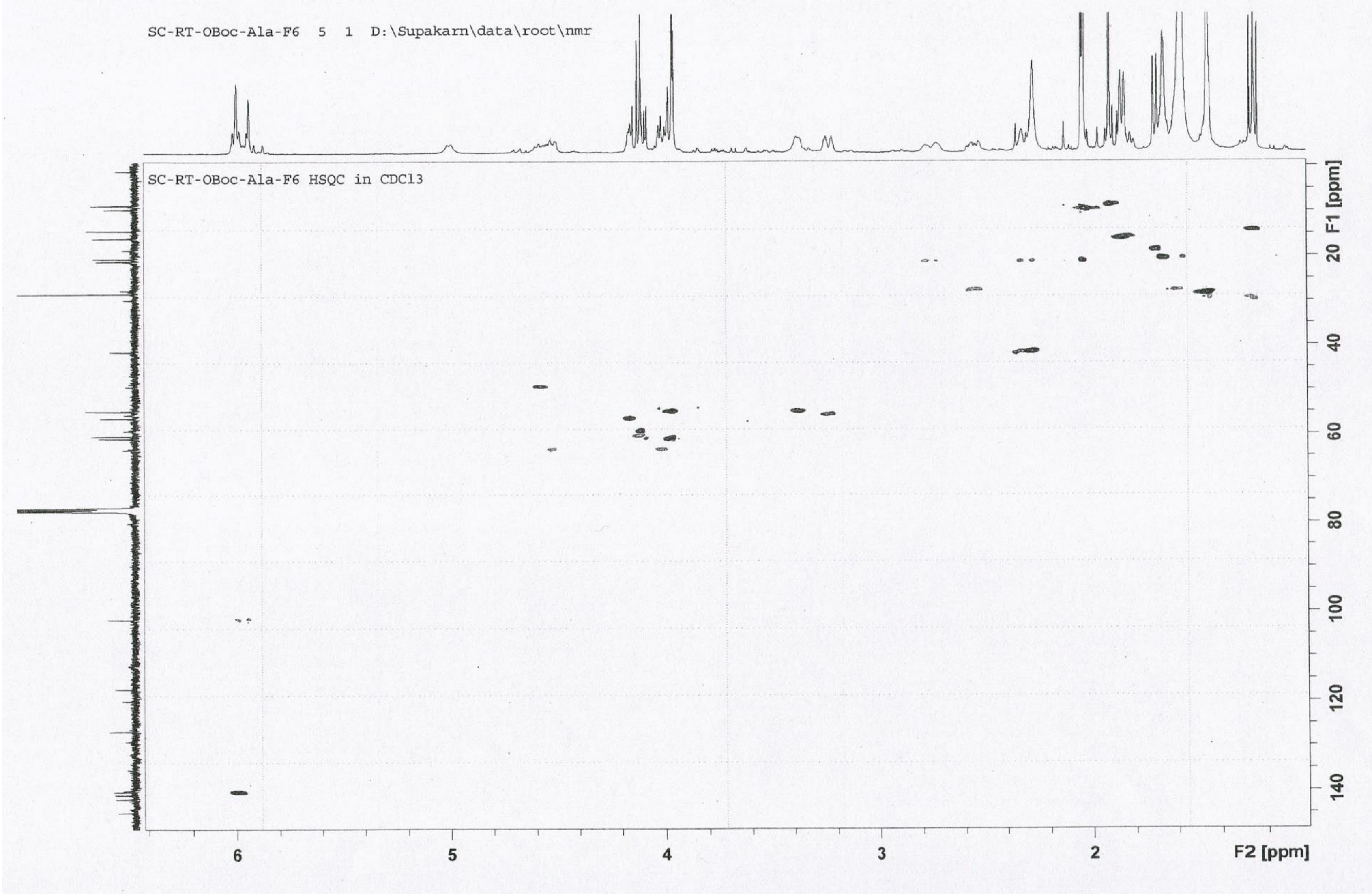

Figure S4. HSQC (400 MHz) spectrum of 5-*O*-(*N*-Boc-L-alanine)-renieramycin T in $CDCl_3$

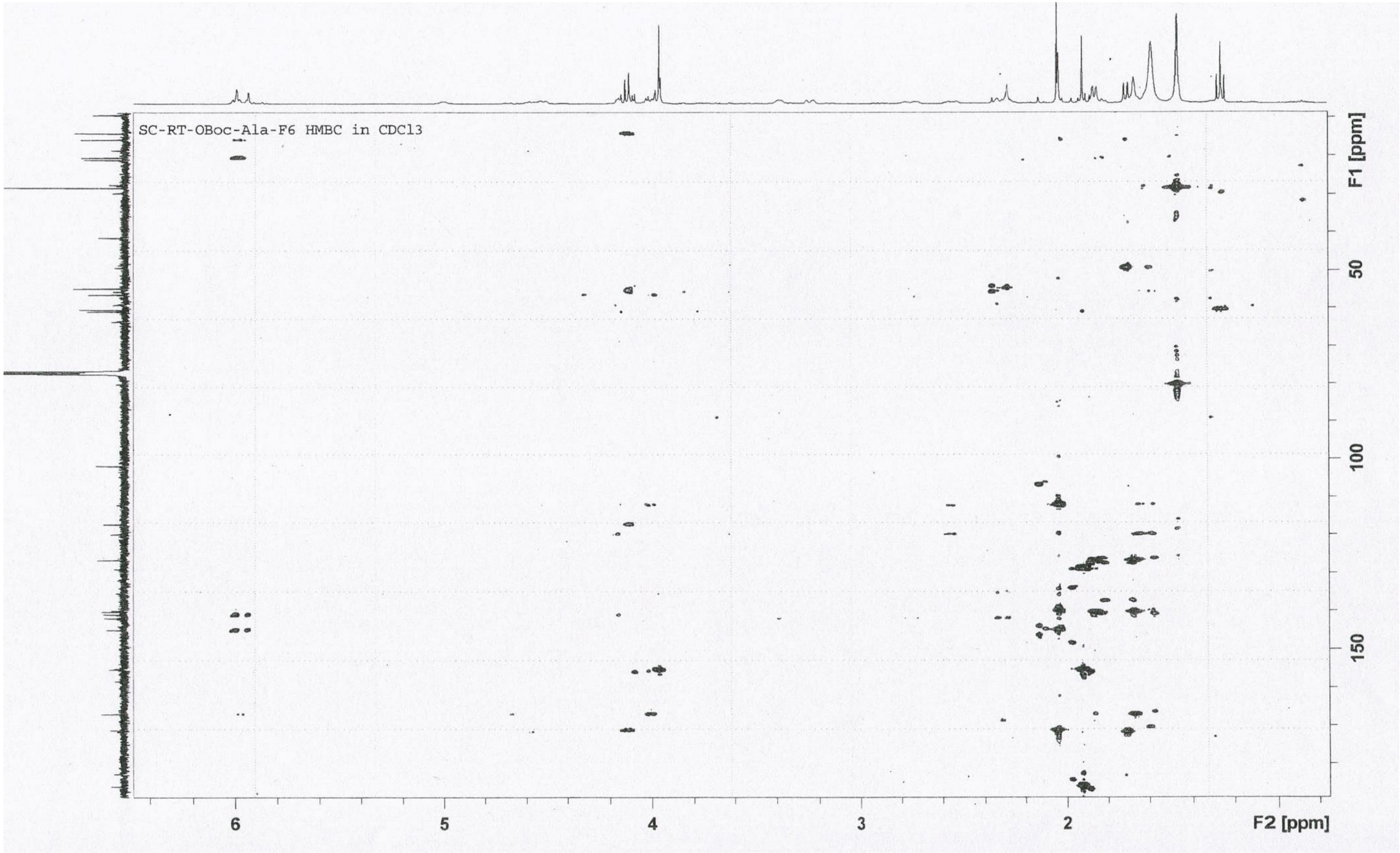

Figure S5. HMBC (400 MHz) spectrum of 5-*O*-(*N*-Boc-L-alanine)-renieramycin T in $CDCl_3$
